# Culturally adapting internet- and mobile-based health promotion interventions might not be worth the effort: a systematic review and meta-analysis

**DOI:** 10.1038/s41746-022-00569-x

**Published:** 2022-03-23

**Authors:** Sumeyye Balci, Kerstin Spanhel, Lasse Bosse Sander, Harald Baumeister

**Affiliations:** 1 Department of Clinical Psychology and Psychotherapy, Institute of Psychology and Education, Ulm University, Lise-Meitner-Str. 16, D-89081 Ulm, Germany; 2 Department of Rehabilitation Psychology and Psychotherapy, Institute of Psychology, University of Freiburg, Engelbergerstr. 41, D-79085 Freiburg, Germany

**Keywords:** Human behaviour, Public health, Psychology, Science in culture

## Abstract

Health promotion interventions offer great potential in advocating a healthy lifestyle and the prevention of diseases. Some barriers to communicating health promotion to people of certain cultural groups might be overcome via the internet- and mobile-based interventions (IMI). This systematic review and meta-analysis aims to explore the effectiveness of culturally adapted IMI for health promotion interventions among culturally diverse populations. We systematically searched on Cochrane Central Register of Controlled Trials (CENTRAL), EbscoHost/MEDLINE, Ovid/Embase, EbscoHost/PsychINFO, and Web of Science databases in October 2020. Out of 9438 records, 13 randomized controlled trials (RCT) investigating culturally adapted health promotion IMI addressing healthy eating, physical activity, alcohol consumption, sexual health behavior, and smoking cessation included. From the included studies 10,747 participants were eligible. Culturally adapted IMI proved to be non-superior over active control conditions in short- (*g* = 0.10, [95% CI −0.19 to 0.40]) and long-term (*g* = 0.20, [95% CI −0.11 to 0.51]) in promoting health behavior. However, culturally adapted IMI for physical activity (*k* = 3, *N* = 296) compared to active controls yielded a beneficial effect in long-term (*g* = 0.48, [95%CI 0.25 to 0.71]). Adapting health promotion IMI to the cultural context of different cultural populations seems not yet to be recommendable given the substantial adaption efforts necessary and the mostly non-significant findings. However, these findings need to be seen as preliminary given the limited number of included trials with varying methodological rigor and the partly substantial between-trial heterogeneity pointing in the direction of potentially useful culturally adapted IMI which now need to be disentangled from the less promising approaches.

PROSPERO registration number: 42020152939

## Introduction

Health promotion interventions are an effective way of disease prevention and improving overall health^1,2^. Well-established approaches focus amongst others on healthy eating, exercising, avoiding excessive use of alcohol, quitting smoking, and sexual health behaviors, such as condom use^3,4^. Promoting these health behaviors on a large scale by using health promotion interventions might be a promising mean for reducing the burden of disease^5–7^. However, these intervention offers do not always reach or fit populations equally between and within countries^8^.

The reach of these interventions could be expanded globally via the internet^9^ using the Internet- and mobile-based interventions (IMI)^10–14^. IMI offers time and place flexibility, potential cost-effectiveness, and scalability without necessarily losing effectiveness^15^. Thereby, IMI could reach a diverse group of people including minorities and people of a cultural background that is not yet well covered by the established health care systems^16–19^. However, IMI also come with some substantial limitations, most prominently risk of low adherence and uptake^20,21^. In order to tackle these issues, population-related factors such as the needs and expectations of the users should be taken into account^15^. Tailoring the intervention content and delivery method to the target group’s culture can thus be a means for increasing engagement and effectiveness^22^.

Researchers in the field of health promotion are encouraged by WHO to approach their practice and research considering diverse human experience and intersectionality of various factors, including culture, gender, immigration status, and ethnicity, which may be related to lower physical and mental health^23–25^. In order to offer an acceptable and relevant health service to people of a certain cultural background, health promotion interventions could be developed from scratch with cultural sensitivity or a less resource-consuming way can, for example, be tailoring an already existing intervention for specific cultural groups^26^. This process is defined as cultural adaptation^27^. Cultural adaptation could be adopted on surface structure modifications (pairing materials and messages to apparent features of the target population such as language) or deep structure modifications (concerning intersecting effects of social, cultural, and historical variables on the target behavior)^28,29^. Culturally adapted face-to-face interventions are shown to be effective in smoking cessation^30,31^, health education, and healthy eating^32^. Moreover, IMI developed for ethnic minorities and underserved populations are also shown to be accepted and effective in the promotion of various health behaviors such as physical activity^33^ and healthy eating^16^.

Previous reviews included IMI for ethnic minority and historically underserved populations in developed countries^22,34–36^. However, none of them specifically examined culturally adapted IMI for health promotion. The number of studies exploring the development and dissemination of culturally adapted health promotion IMI is increasing and thus a systematic review and meta-analysis seems timely. Hence, this review aims to systematically identify culturally adapted IMI on health promotion and explore their effectiveness among populations that is different from the original intervention’s target group.

## Results

All the predefined characteristics of included articles are presented in Table 1.

**Table 1:** Characteristics of included articles.

1st author (year)	Country	Sample	Sample size E: experiment group/C: control group	Gender Female (%)	Mean age (SD)	Dropout rate at post-assessment (%)	Website vs. Mobile	Duration/ No. modules	Post randomization follow up in months	Comparison	Outcome	Outcome measures
Augustson et al.^37^	China	Adults smokers	E: 4000 C: 4000	3.6	–	73	SMS	6 weeks	1,3 & 6 M	Active control group The Low-Frequency Text Contact (LFTC) received 1 text message a week, for the 6-week intervention period	Smoking cessation	Smoking status was based on past-7-day abstinence self-reported via text message
Bender et al.^38^	USA	Individuals diagnosed with Type 2 Diabetes	E: 22 C: 23	62	57.6 (9.8)	2.5	App/ Social media	26 weeks	3 & 6 M	Active control group receives only Fitbit accelerometer and training for daily wear.	Physical activity	Step count via the Fitbit Zip. (accelerometer data)
Bowen et al.^39^	USA	Students (6th to 12th graders)	E: 64 C: 49	53	14.6	9	Website	6 weeks	1 M	Waitlist control	Smoking cessation	Smoking status based on “A Smoking Prevention Interactive Experience (ASPIRE)” instrument
Brito Beck da Silva et al.^40^	Brazil	Students (7^th^ to 9^th^ grade)	E: 428 C: 467	46	14.49 (1.42)	30	Website	16 weeks	12 M	Waitlist control	Healthy eating	BMI
Cruvinel et al.^41^	Brazil	Adult smoker post-discharge patients	E: 44 C: 22	45	47.7 (11.5)	10	Mobile/ SMS	2 weeks	1&3 M	Treatment as usual includes educational materials, brief intervention (BI), and access to NRT (adhesive patch and gum)	Smoking cessation	Smoking status of smokers (cigarettes a day) and self-reported 7-day point prevalence abstinence post- randomization.
Duan et al.^42^	China	University students	E: 270 C: 223	60	19.3 (1.07)	45	Website	8 weeks	2&3 M	Waitlist control	Physical activity & Quality of life	Chinese short version of the International Physical Activity Questionnaire (IPAQ-C) & Hong Kong version of the WHO’s Quality of Life-BREF questionnaire
Fortmann et al.^43^	USA	Individuals diagnosed with Type 2 Diabetes	E: 63 C: 63	75	48.43 (9.8)	10	SMS	26 weeks	3&6 M	Treatment as usual (standard diabetes care provided by primary care providers at the clinic and group Diabetes self-management education- use of these services based on patient and physician’s initiative)	Healthy eating	BMI
Kurth et al.^44^	USA	HIV + individuals	E: 226 C: 207	55	47.8	8	Website	52 weeks	3,6 & 9 M	Active control group (received computer-based audio-narrated risk assessment, which included questions about sexual risk behaviors, substance use, mental health, social support, partner status and disclosure, ART regimen and adherence in last 7 and 30 days, and side effects.)	Sexual health behavior	sexual transmission risk behaviors (lack of condom use with either a main or another partner)
Larsen et al.^45^	USA	Adult male	E: 22 C: 24	0	43.04 (10.67)	6	SMS	24 weeks	6 M	Active control group (wellness control group received two SMS weekly throughout the study and publicly available print-based materials on health topics different from physical activity)	physical activity	Minutes/week of moderate to vigorous PA (MVPA) measured by accelerometers
Lau et al.^a^ ^46^	Hong Kong	Students aged between 12-16 years old	E:13 C: 16	49	13.7	not reported	SMS	4 weeks	1 M	No treatment	physical activity	Self-reported physical activity via PAQ-C (Physical activity questionnaire)
Marcus et al.^47^	USA	Inactive adult Latinas	E: 104 C: 101	100	39.20 (10.47)	not reported	Website	26 weeks	6 M	Active control group (wellness contact, receive access to a Spanish language website with information on health topics different from physical activity)	physical activity	Minutes/week via 7-day Physical Activity Recall and accelerometers.
Montag et al.^48^	USA	American Indian/ Alaska Native women	E: 113 C:134	100	28.6	6	Website	20 min	1,3 & 6 M	Treatment as usual (get access to displayed educational brochures about health apart from FASD (fetal alcohol spectrum disorders) related information in the various waiting areas)	Alcohol consumption	Level of alcohol consumption (number of drinks per week)
Peiris et al.^49^	Australia	Current Aboriginal smokers (>16 years old)	E:25 C: 24	78	42 (14)	6	Mobile App	53 weeks	1&6 M	Active control group (encouraged to use any other smoking cessation service or support and were offered Quitline and local ACCHS (Aboriginal Community Controlled Health Services) contact numbers)	Smoking cessation	Smoking status, self-reported abstinence

^a^Provided three intervention groups versus a control group comparison, we used the intervention group which had the most exposure to the intervention as a comparator.

### Study selection

We identified a total of 20,012 records. After screening titles and abstracts and full-texts, 13 studies were included in the quantitative analyses. The main characteristics of the studies are outlined in Table 1. During the full-text screening, 38 studies were excluded due to lack of cultural adaptation, 29 studies were concerning culturally sensitive interventions, 13 studies did not report relevant outcome data, eight studies were no original articles, four studies were not RCTs, five were excluded due to other reasons (i.e. novel interventions, study protocols, non-English full-text) and one due to a differing health promotion topic.

### Study characteristics

The 13 RCTs included in this review comprise a total of *N* = 10,747 randomized participants, mostly adult populations (*N* = 9710). Trials were conducted between 2012 and 2020^37–49^. The mean age of the participants varied from 14 to 57 (see Table 1). Primary studies focused on smokers (*k* = 3, *N* = 2546), individuals diagnosed with Type 2 diabetes (*k* = 2, *N* = 171), and HIV + individuals (*k* = 1, *N* = 433).

Four studies dealt with smoking cessation^37,39,41,49^, two with both healthy eating and physical activity^38,42^, three with physical activity only^45–47^, two with healthy eating only^40,43^, one with sexual health behavior^44^, and one with alcohol consumption^48^.

Four studies provided follow-up data on short-term effectiveness (one to five months follow-up)^39,41,42,46^, three studies on long-term effectiveness (six to 12-months follow-up)^40,45,47^, six studies provided follow-up data on both assessment points.

Seven studies were conducted in the USA^38,39,43–45,47,48^, two in China^37,42^, two in Brazil^40,41^, one in Hong Kong^46^, and one in Australia^49^.

The cultural adaptation was based on a theory or a guideline in three studies^38–40^. Eight studies based their cultural adaptation on a formative research/pilot study or expert review^37,41,42,44,45,47–49^. Two studies did not provide information regarding the basis of cultural adaptation^43,46^. In terms of alterations of the intervention content, four studies incorporated both surface and deep structure changes^38,40,43,45^, while nine studies used surface structure changes only^28^. Details of the culturally adapted and original interventions are presented in Table 2.

**Table 2:** Summary of culturally adapted and original IMI.

1st author (year)	Name	Language	Target group	Ethnicity	Health promotion	Cultural adaptation theory	Cultural adaptation components
	original IMI	original IMI	original IMI	original IMI	original IMI		
	adapted IMI	adapted IMI	adapted IMI	adapted IMI	adapted IMI		
Augustson et al.^37^		English	General population	US American	Smoking cessation	Expert review, focus groups	Language, context adaptation
	Change to Quit China	Chinese	General population	Chinese	Smoking cessation		
Bender et al.^38^	Diabetes Prevention Program (DPP)	English	Type 2 Diabetes patients	American	Healthy eating/physical activity	Bender & Clark (2011)’s theory^107^	Content (Filipino food photos), delivery (involvement of family members to the office visits) language
	PilAm Go4Health	English	Type 2 Diabetes patients	Filipino	Healthy eating/physical activity		
Bowen et al.^39^	SmokingZine	English	General population (adolescent)	Canadian	Smoking cessation	Based on a guideline from Wisdom2Action	Images, context
	-	English	General population (adolescents)	American Indian	Smoking cessation		
Brito Beck da Silva et al.^40^	StayingFit	English	General population	US American	Healthy eating/physical activity	Based on Barrera et al (2013)^108^ and Castro et al (2015)^109^	Language, cultural standards, meanings, and values added
	StayingFit Brazil	Portuguese	General population (adolescents)	Brazilian	Healthy eating/physical activity		
Cruvinel et al.^41^	-	English		US American	Smoking cessation	Formative research	Language, information from the Brazilian smoking cessation treatment guideline
	TXT	Portuguese	Hospitalized smokers	Brazilian	Smoking cessation		
Duan et al.^42^	-	-	General population	US, Germany and Netherlands	Healthy eating/physical activity	Formative research	Language, content
	-	Chinese	General population (students)	Chinese	Healthy eating/physical activity		
Fortmann et al.^43^	Staged Diabetes Management (SDM) & Dulce Project	English	General population	US American	Healthy eating/diabetes management	Based on face-to-face intervention project Dulce^110^	Language, cultural beliefs that interfere with optimum self-management, shortened content, motivational messages
	Dulce Digital	English and Spanish	Type 2 Diabetes patients	Hispanic	Healthy eating		
Kurth et al.^44^	CARE +	English	HIV + patients	US American	Sexual health behavior	The local expert advisory panel, usability testing	Content (Language), expert suggestions
	CARE + Spanish	Spanish	HIV + patients	Latino	Sexual health behavior		
Larsen et al.^45^	Seamos Saludables	Spanish	General population	US American	physical activity	Formative research and pilot (qualitative interviews)	Language adaptation, the content of the SMS, and printed materials
	Activo	Spanish	General population (men)	Latino	physical activity		
Lau et al.^46^	-	English	General population	US American/Canadian	physical activity	NA	Language, content (colloquial dialogue for adolescents)
	-	English, Dutch, Turkish	General population	Hong Kong Chinese	physical activity		
Marcus et al.^47^	-	English	General population	US American	physical activity	Focus groups	Cultural and linguistic adaptation, culturally adapted content and support specifically for Latinas, flexible scheduling for assessment meetings, reimbursement for travel and childcare
	Pasos Hacia la Salud	English	General population (women)	Latina	physical activity		
Montag (2015)	e-CHUG	English	General population	US American	Alcohol consumption	Focus groups	Content (pictures, logo, color of the layout, example characters, myths) - added video (verbal tradition)- language (not a translation but wording and simplifying)
	eCHECKUP TO GO	English	General population (women)	American Indian/Alaska Native (AIAN)	Alcohol consumption		
Peiris et al.^49^	QuitTxt	English	General population	Australian	Smoking cessation	Formative research with the expert user group	Adaptation of the content and tone of the messages based on the attitudes of the target group towards smoking
	Can’t Even Quit’	English	General population	Australian/Aboriginal/Citizen of Torres Strait Island	Smoking cessation		

### Risk of bias of in included studies

**Fig. 1:**
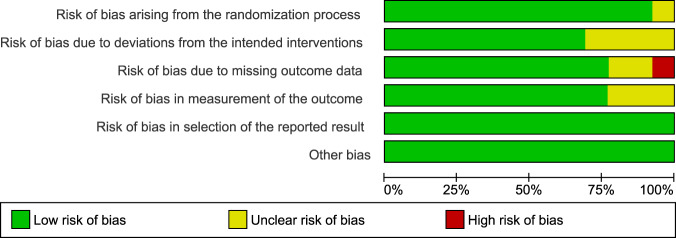
Risk of bias summary. Reviewers’ judgments about each risk of bias item for each included study.

**Fig. 2:**
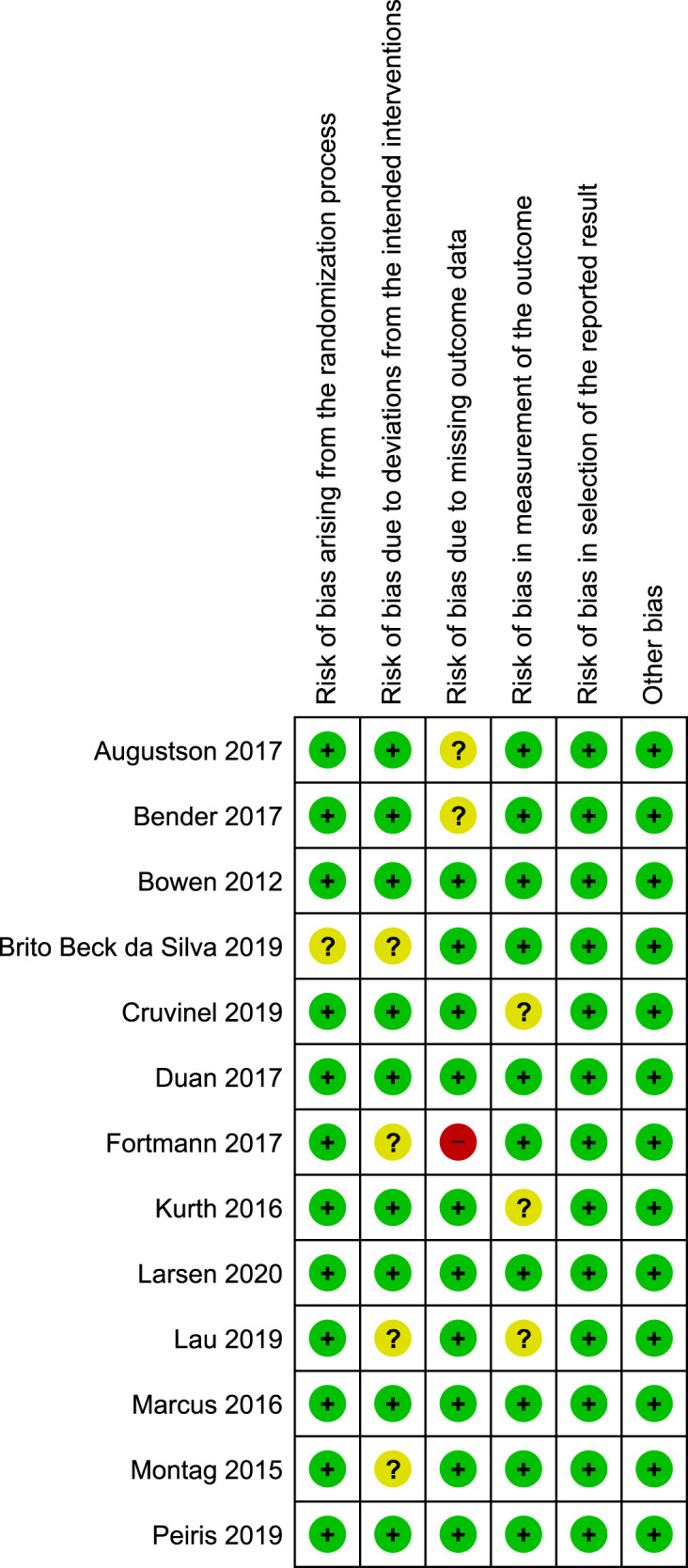
Risk of bias graph. Reviewers' judgments about each risk of bias item presented as percentages across all included studies.

The risk of bias assessment of the included studies is presented in Figs. 1, 2. The interrater reliability suggested substantial agreement between the raters, *κ* = 0.79. Five studies were assessed to have a low risk of bias, five studies had some risk of bias and three studies were rated to have a high risk of bias. Four studies were assessed to have some risk of bias due to deviations from the intended intervention^40,43,46,48^, three studies were assessed to have some risk of bias in the measurement of the outcome domain^41,44,46^. Two studies^37,38^ were assessed to have some risk of bias due to missing outcome data, one study^43^ had a high risk of bias in this domain. One study was assessed to have some risk of bias arising from the randomization process^40^. This study had an unequal number of clusters, which resulted in significant baseline differences in primary outcomes.

### Effectiveness

Nine studies that provided data to calculate standardized mean difference effect sizes were pooled. Individual data points are presented in Supplementary Table 1. Four studies concerning smoking cessation did only provide dichotomous outcomes. We conducted separate meta-analyses for the different areas of health promotion (physical activity, smoking cessation), in the case of at least three studies reported on the same outcome. Since we included only nine studies in the analysis, we refrained from exploring the publication bias via a funnel plot^50^. Following, we report on pooled effectiveness across health promotion domains.

**Fig. 3:**
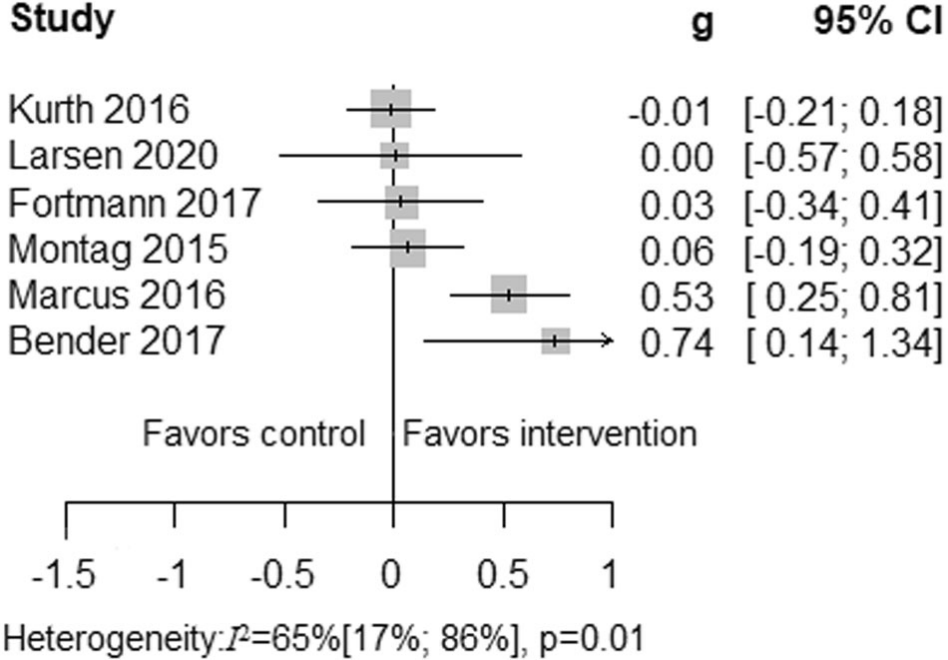
Summary of culturally adapted IMI of health promotion vs. active controls in the long-term. Due to substantial heterogeneity among the culturally adapted IMI of health promotion vs. active controls in long-term meta-analytical pooling did not perform.

**Fig. 4:**
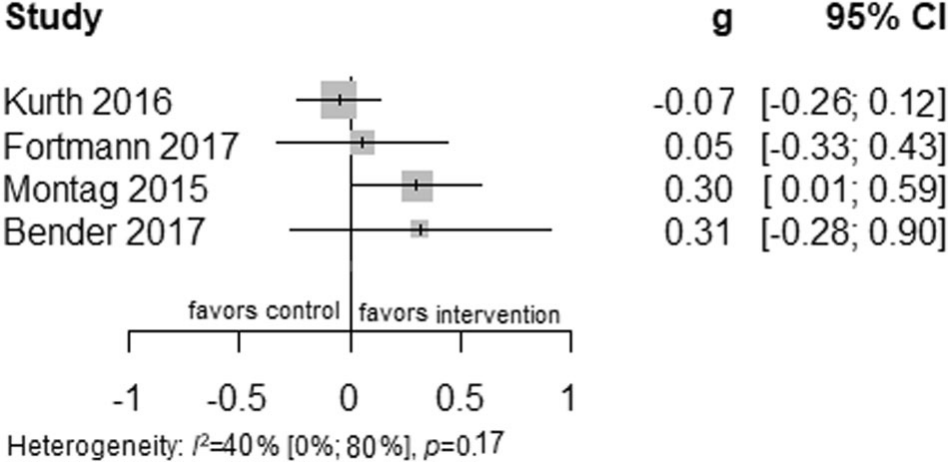
Summary of culturally adapted IMI of health promotion vs. active controls in the short-term. A summary plot of effect sizes of four studies of culturally adapted IMI of health promotion vs. active controls in short-term are presented.

The meta-analysis of six studies^38,43–45,47,48^ examining any long-term health promotion interventions revealed that culturally adapted IMI of health promotion were not superior to active control conditions in the long-term. In addition, four studies^38,43,44,48^ that provided short-term follow-up data were not superior to active control conditions in improving health behavior outcomes. Due to substantial heterogeneity, the results are reported descriptively, see Figs. 3, 4.

**Fig. 5:**
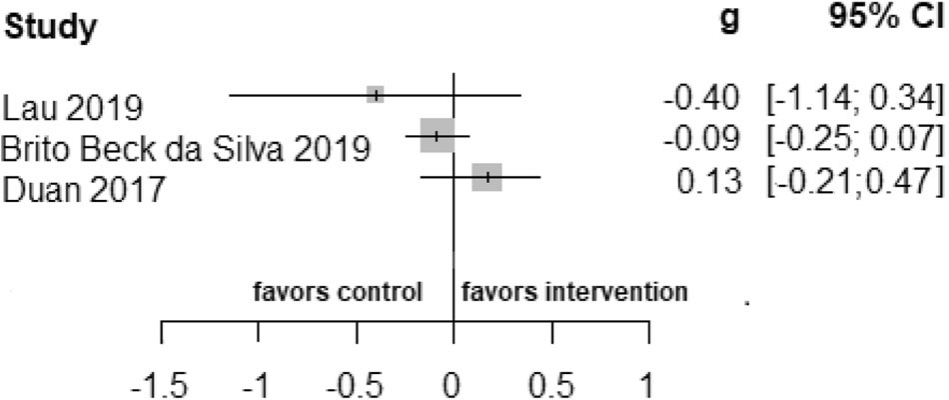
Summary of culturally adapted IMI of health promotion vs. passive controls. Due to few numbers of studies (two studies reported data in the long-term, two in the short-term, while one study reported dichotomous outcome) comparing culturally adapted IMI to a passive control group, meta-analytic pooling did not perform.

Comparisons with passive control groups could not be pooled given the low number of studies (*k* = 4), see Fig. 5. One study^39^ reported only Odds ratios ([OR], 1.13; 95% CI 0.18 to 7.04).

Due to a small number of studies (*k* = 9), we did not perform the predefined subgroup and sensitivity analyses.

### Effectiveness of culturally adapted IMI of physical activity

**Fig. 6:**
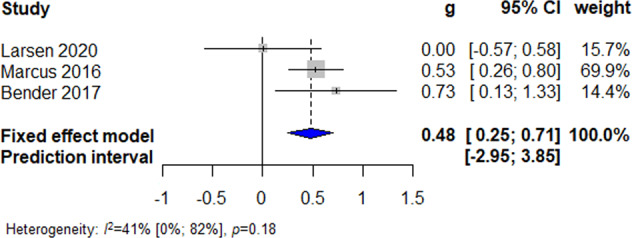
Fixed effects meta-analysis of culturally adapted IMI for physical activity vs. active control conditions. Forest plot presenting fixed effects meta-analysis of culturally adapted IMI for physical activity vs. active controls.

Five studies reported physical activity outcomes, four of which provided data via accelerometers/pedometers^38,45,47^ and one via a self-reported questionnaire^46^. Pooling the three studies with active control conditions resulted in a small significant long-term effect favoring culturally adapted IMI (*N* = 296; *g* = 0.48; 95% CI 0.25 to 0.71; *I*^2^ = 41%; fixed effect), see Fig. 6.

### Effectiveness of culturally adapted IMI of smoking cessation

**Fig. 7:**
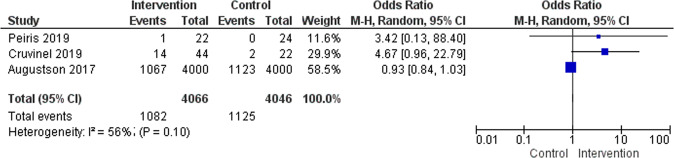
Summary of culturally adapted IMI for smoking cessation vs. active controls in short-term. Three studies reported smoking cessation outcomes measuring short-term abstinence at the end of the intervention vs. active controls are presented on the forest plot.

Three RCTs (*N* = 8,112) reported smoking cessation outcomes measuring short-term abstinence at the end of the intervention versus active controls. The meta-analysis findings of these studies were not significant (Odds Ratio [OR], 1.75; 95% CI 0.51 to 6.05, *I*^2^ = 56%), see Fig. 7. The number of included studies was small (*k* = 3) and the effect size was mainly based on one large-scale study with *N* = 8000 participants^37^.

### Effectiveness of culturally adapted IMI of healthy eating, sexual health behavior, and alcohol consumption

Fewer than three studies reported on sexual health behavior^44^, alcohol consumption^48^, and healthy eating^40,43^. Only one generic outcome, health-related quality of life, was reported in one trial^42^. These studies were not eligible for pooling due to the number of trials in the respective outcome but reported in Figs. 3–5.

## Discussion

To our knowledge, this is the first systematic review and meta-analysis that assessed the effectiveness of culturally adapted IMI on health promotion among populations which the intervention was originally not developed for. Our results suggested that culturally adapted IMI are not more effective in promoting health behaviors than active conditions in short- (*g* = 0.10) and long-term (*g* = 0.20), respectively. When regarding health promotion topics separately, health promotion of physical activity resulted in a small to moderate effect favoring culturally adapted IMI over active control conditions in the long-term (*g* = 0.48). This is in line with a previous umbrella review of health promotion IMI for minority and historically underserved populations, which, however, not exclusively included culturally adapted IMI^22^. Similarly, another meta-analysis of IMI concerning physical activity did highlight the superiority of IMI over a control group or no-treatment condition, without a specific focus on culturally adapted interventions^14^. No other significant effect was revealed for the other addressed health promotion topics.

Subgroup analyses aiming to detangle the substantial between trial heterogeneity were not feasible. Heterogeneity among the included studies was moderate to substantial, *I*^2^ ranged from 0.36 to 0.66. Prior research points to differential effects on culturally adapted health promotion interventions in terms of different populations (age and ethnicity^51^), different intervention features (professional vs. non-professional provider)^32^, intervention duration and follow-up times in culturally adapted face-to-face interventions^52^, interventions focusing on general population groups^53^, different methodological decisions (RCT methodology use, different control groups, e.g. tailored website, no-treatment controls^53^) and cultural adaptation contents (inclusion of social support and/or family members^51^, integrating cultural beliefs and values^54^). There might be further possible explanations for between-study heterogeneity, and future research needs to provide a better understanding of the impact such factors have on the effectiveness of culturally adapted IMI for health promotion. The present findings are inconsistent with previous meta-analyses on IMI in several ways. Among western populations, these meta-analyses yielded positive effects favoring IMI compared to a waitlist and/or active controls (e.g. other internet-based or face-to-face interventions): for smoking cessation and abstinence outcome^10,55^, which is maintained at 12-month follow-up^56^ and higher effects achieved with the use of tailored messages^10,13^; healthy eating^57–59^ and weight loss^60^; sexual health behavior promotion^61^ and regarding HIV prevention and condom use^62^. Another meta-analysis found significant positive effects of tailored (based on personal relevance) web-based interventions on health behaviors compared to non-tailored web-based interventions^53^ and a different meta-analysis of SMS-based interventions on various health behavior outcomes suggested that targeted and tailored (based on demographic and psychosocial factors) SMS yielded larger effect sizes, especially for physical activity interventions (*g* = .51), which yielded a similar effect size to our results (*g* = 0.48)^10^. Most surprisingly in this context is our null-finding regarding the effectiveness of culturally adapted IMI compared to waitlist control conditions, which are known to provide a rather upper benchmark of the benefit of interventions^63^, usually associated with significantly larger between-group effect sizes in IMI for health promotion as well^10,13,64^. Although some of the meta-analyses mentioned above concern tailored intervention contents, none of the above-mentioned meta-analyses were specifically examining culturally adapted IMI. Therefore, our results cannot be easily compared with prior meta-analyses. However, if culturally adapted IMI for health promotion are not effective at all, even when compared to waitlist controls, we might need to challenge the idea of providing culturally adapted IMI to populations for which the intervention was originally not developed for at large and examine whether IMI developed with cultural sensitivity are effective in the same target groups. Hence, explanations for this surprisingly limited effectiveness seem warranted.

One possible explanation of our results might be related to the quality of cultural adaptation of the interventions. The cultural adaptation processes were rarely well defined in the included studies. Therefore, it was not clear whether aspects of cultural adaptation were appropriate. In addition, the high dropout among included studies could be an indicator of cultural adaptation not working as intended by the researcher. Moreover, only three studies based their cultural adaptation process on a theory, which might contribute to its quality. A tested theory of cultural adaptation of IMI is missing. However, there are guidelines developed for culturally adapting face-to-face interventions^28,65^ and researchers could implement these guidelines when adapting an IMI^66^. A recent taxonomy of cultural adaptation of IMI for mental disorders serves as a basis for future cultural adaptations of IMI^67^. Adopting a theoretical basis in intervention development is suggested to result in higher effects, as was shown in a meta-analysis^14^. However, this could not be shown in our results: only one out of the three IMI that utilized a theory of cultural adaptation resulted in an improvement in physical activity outcome^38^. In the future, culturally adapted intervention studies should consider supporting cultural adaption with an established theory and report the adaptation process in more detail to lead prospective cultural adaptations and replications. In the process of cultural adaptation, some of the included studies sought expert reviews, focus group feedback, and conducted a pilot study, and at least altered one aspect of the intervention. However, the majority of the changes were regarded as taking place only at the surface structure^28^, which might be one reason for the limited impact of culturally adapted IMI shown in the present review. Surface structure changes aim at improving feasibility while deep structure changes target program’s effect for the participants^28^. Implementing deep structure changes involves core cultural values of a certain population, such as beliefs towards a health issue, or performing a behavior as a member of gender identity. A meta-analysis of face-to-face culturally adapted health interventions showed that incorporating family members and religious values, which are considered as deep structure changes, in the intervention was related to improvements^31^. Future research should consider exploring surface versus deep structure alterations on the effectiveness of IMI e.g. in the framework of dismantling and additive clinical trial designs aiming at detangling active components and mechanisms of change of the respective interventions^68–71^.

Moreover, culturally adapting interventions is not free from criticism. It should be taken into account that the majority of the interventions developed in the fields of psychology and behavioral medicine are for a rather homogeneous group (white, educated, middle to high socioeconomic status) but not representative for the majority^19,72^. In the cultural adaptation process, the same intervention is often altered to cater to the needs of a different group of people that are non-white, occasionally less educated, and/or bilingual. This process could be seen as a form of assimilation for the target group because even the topic of the intervention might also be representative of western, white, educated humans^1^. Therefore, in order to avoid these issues, the first step of cultural adaption might include approaching the problem and defining it with the cultural sensitivity of its target group^73^. In addition, people of a certain cultural background are not homogenous within themselves, each member’s experience is affected by intersecting factors^74^. Therefore, it might be more complicated than often expressed to adapt an intervention for a cultural group^75^. One possible solution might be to invest in adapting interventions to cultural specifics of the users, e.g. based on user needs assessments^76^ or community leaders’ input^77^. Another solution, especially for migrant/immigrant populations could be developing interventions based on the target groups’ acculturation levels, i.e. a process on a spectrum of either orientation to the host culture or maintaining the native culture^1,76^. It seems also worthwhile to pay attention to intersecting factors that might influence a member of a cultural group, namely gender and literacy. However, we first need to establish whether to culturally adapt health promotion IMI at all. The present findings at least suggest—except for physical activity IMI—a non-favorable cost-benefit ratio, a result that still is in need of stronger evidence.

Another topic of relevance to our findings is the reach, uptake, and intervention adherence of culturally adapted IMI. Even if such IMI would be effective, they still need to be used in order to exploit their full potential. Although internet technologies are widely used globally^78^, there are still barriers to utilizing these technologies, which cause inequalities in accessing the internet and mobile technologies and comprehending health information^16,79,80^. Moreover, pure mobile-based interventions are seemingly less effective than internet-based or combined interventions^81–83^, which might also affect for instance minority populations, where they are more likely to use a smartphone to access the internet than non-minority populations where multiple device (e.g. tablets, desktops) ownership is common^84^. To increase adherence, multimodal content and guidance (direct contact with the provider)^85^ might be useful via diminishing issues of health literacy, motivational and volitional aspects, and the digital divide^71,86,87^. These aspects might be particularly important in people of certain cultural backgrounds living in a high-income country, people living in low-income countries, and/or vulnerable populations, such as immigrants, given the limited representation of many of these populations in the health research, and high rates of drop-out^86,88–91^.

This review has some limitations. First, we included interventions concerning only five prominent areas of health promotion thus results might not be generalizable to other health promotion domains. Second, the definition of cultural adaptation varies, and our operationalization of culturally adapted interventions resulted in the exclusion of studies that investigated IMI that were developed newly in a culturally sensitive way. Comparison of culturally adapted vs. culturally sensitive interventions is an interesting further research topic. Moreover, comparing culturally adapted versus culturally sensitive IMI might present insights into whether it is worthwhile to develop a novel IMI for a group or adapt an already existing one. Third, we were able to pool data from only 13 studies, which further limit the generalizability of our results. Due to this limited evidence base, analyses were restricted to the main research questions while subgroup analyses were not feasible yet. Future updates might allow for exploring the between-study heterogeneity highlighted in the present review, while the findings reported here could guide researchers in what to examine next. In this context, we suggest adding generic outcomes such as health-related quality of life, daily functioning, or self-efficacy to domain/disease-specific outcomes to allow for cross-trial cross health promotion domain comparisons in the future. Fourth, although the studies included in this meta-analysis were culturally adapted, the adaptation process was rarely well defined. Fifth, only three out of 13 studies used a theory to adapt the intervention. And last, none of the studies were comparing culturally adapted IMI to non-adapted IMI. This creates a difficulty to draw any firm conclusions about the differential effectiveness of culturally adapted interventions. Despite these limitations, this meta-analysis had some strengths. To our knowledge, this is the first meta-analysis concerning culturally adapted health promotion IMI. Moreover, this a-prior registered systematic review included mostly scientifically sound RCTs from a broad sample and health behavior topics. A summary of recommendations for future research is represented in Box 1.

Box 1 Recommendations for future research

1. Improve evidence on whether culturally adapted IMI for health promotion are indeed not effective at all, even when compared to passive control conditions.
2. Provide a better understanding of the impact of population and intervention characteristics on the differential effects of culturally adapted IMI for health promotion.
  If culturally adapted IMI are effective at least compared to passive control conditions (see 1.) and/or at least with regard to some subgroups (see 2.):
3. Improve evidence on whether culturally adapted IMI are effective compared to active control conditions.
4. Particularly compare culturally adapted IMI with the respective non-adapted versions or simple language translations of the IMI.
  Ultimately, the substantial effort necessary for adapting IMI culturally might only be justified in case of clinically significant superiority of the culturally adapted versions compared to active controls. In case this is given (see 3./4.)- at least with regard to some subgroups (see 2.):
5. Examine active components and mechanisms of change of these effective culturally adapted IMI. Particularly provide a better understanding of the impact of surface and deep structure changes on intervention adherence and effectiveness.
6. Examine ways of improving reach, uptake, engagement, and intervention adherence for the effective culturally adapted IMI.
7. Develop evidence-based recommendations and guidelines for adapting IMI culturally delineated from the effective culturally adapted IMI.

## Conclusion

Based on the present findings, culturally adapted IMI might not be superior compared to control conditions in the short- and long-term, except for physical activity. Although they might exhibit a more attractive health offer to their target group, their usefulness is questionable or at least need further examination. Thereby, it might be worthwhile to take into consideration intersecting aspects of experiences of people of certain cultural groups regarding health behaviors to assure acceptability and effectiveness when designing interventions and contribute to diminishing health inequalities.

## Methods

### Protocol and registration

This systematic review and meta-analysis has been registered at PROSPERO (Registration number: CRD 42020152939) and follows the format of the PRISMA guideline^92^. Review protocol^93^ described the aim, methodology, and data analysis plan in advance. Changes to study protocol are listed in the supplementary notes.

### Eligibility criteria

Studies were included if they (1) were RCTs, (2) had no treatment, treatment as usual (TAU), placebo, waitlist, or active control conditions, (3) were delivered via the internet, (4) were culturally adapted for a population that differed from the original intervention’s target group, (5) examined a health promotion intervention on healthy eating, physical activity, alcohol consumption, sexual health behavior and/or smoking cessation (6) reported one of the respective health promotion-specific outcomes: body mass index (BMI), time spent exercising, change in condom use, level of smoking, level of alcohol consumption, or one of the following generic outcomes: health-related quality of life and self-efficacy.

### Information sources and study selection

The initial search was conducted in the following databases on 26. August 2019: Cochrane Central Register of Controlled Trials (CENTRAL), EbscoHost/MEDLINE, Ovid/Embase, EbscoHost/PsychINFO, and Web of Science. A combination of keywords (including MeSH terms) indicating culturally adapted IMI for health promotion has been used. The search terms are published in this review’s protocol^93^ and cover comprehensively both the topic of the present review as well as one of a parallel systematic review on culturally adapted IMI for mental health conditions^94,67^. There were no restrictions on the publication date. A search update was conducted on 15 October 2020.

**Fig. 8:**
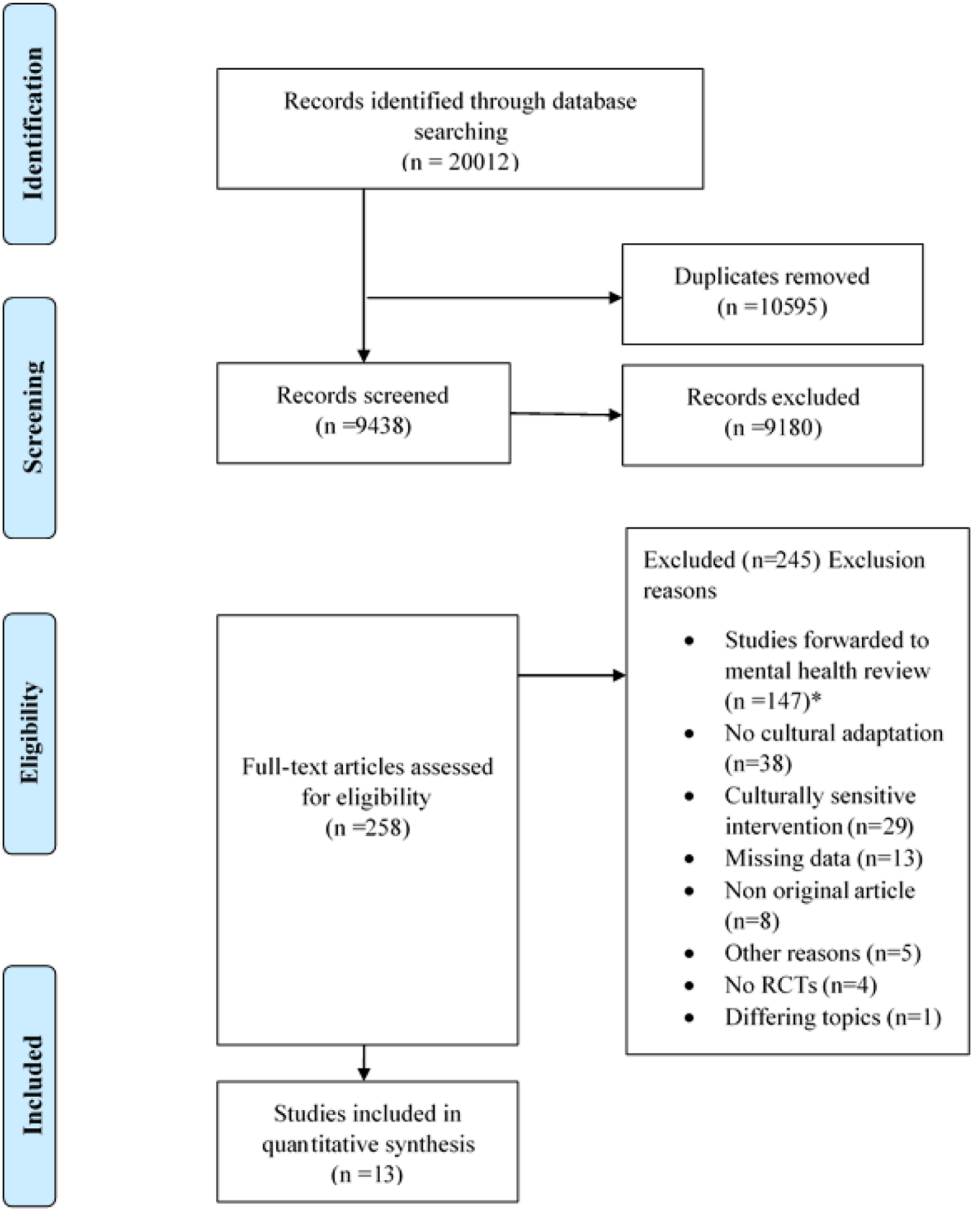
Prisma Flow chart^92^. Study identification, selection, and inclusion represented on the diagram. An asterisk symbol represents a parallel review conducted regarding the culturally adapted internet- and mobile-based interventions concerning mental health.

All search results were merged into Covidence^95^ and duplicates were automatically removed. Two reviewers screened titles and abstracts of the identified articles against the inclusion criteria and selected potentially relevant articles for the full-text screening. Full-text screening has been performed by two reviewers independently, disagreements have been solved by consensus or a third reviewer where needed. The study selection is illustrated in the PRISMA flow diagram (see Fig. 8).

### Data extraction

Data extraction was conducted by two independent reviewers and then extracted data was then checked by a third reviewer. The following data were extracted from the included studies: publication details, study participants (demographics and cultural background, baseline characteristics), study design, study setting, characteristics of the original and culturally adapted intervention, health behavior-specific and generic outcome measures, information regarding cultural adaptation (content, utilization of theoretical or evidence-based components). Behavioral outcomes are defined as: physical activity measured via physical activity minutes per week with accelerometers or self-report questionnaires; healthy eating measured via BMI; alcohol consumption assessed via the level of alcohol consumption; smoking cessation assessed via the level of smoking or the abstinence percentage; sexual health behavior assessed with condom use. Generic outcomes were defined as health-related quality of life and self-efficacy, assessed by means of validated self-report questionnaires. Control conditions were categorized into active (placebo, other health promotion interventions & TAU) and passive controls (waitlist & No treatment). When related information could not be extracted, corresponding authors of the articles were contacted to obtain information. The extracted data was tabulated.

### Risk of bias

Two independent reviewers performed quality assessments with Cochrane Collaboration’s Risk of Bias Tool 2.0^96^. The RoB tool 2.0 has five domains including bias arising from the randomization process, bias due to deviations from intended interventions, bias due to missing outcome data, bias in the measurement of the outcome, bias in the selection of the reported result. A third reviewer solved disagreements following a discussion between the reviewers. The Kappa statistic was used to calculate interrater reliability^97^.

### Meta-analysis

For each study, a standardized mean difference (SMD) and 95% confidence intervals (CI) were calculated with mean scores of intervention and control groups. In order to decrease the bias of small samples, Hedges’ g was calculated^98^. Effect sizes were recoded when higher scores of an outcome assessment indicated worsening results (e.g. BMI and level of alcohol consumption). For continuous outcomes, Hedges’ g and 95% CIs were reported; for dichotomous outcomes, odds ratios and CIs were reported. Random effects model was chosen for analyses due to an expected diversity among IMI of health promotion, sample size, and duration of intervention among studies^99^. Data were pooled to calculate a standardized mean effect size for each outcome and a forest plot with 95% CIs, in the case of at least three studies reporting the respective outcome. Otherwise, results were presented descriptively. Sensitivity analysis was planned to assess the impact of studies with a high risk of bias. Analyses were performed in R package meta and metafor, and Review Manager 5^100–103^. Continuous effect sizes were categorized along with Cohen’s rule of thumb with 0.20 considered a small effect, 0.50 medium effect, and 0.80 large effects^104^. In order to assess publication bias, we planned to conduct funnel plots.

Statistical heterogeneity among studies was analyzed with the *I*^2^ statistics^97,105^. Statistical heterogeneity refers to the variability among effect sizes in a meta-analysis^106^. However, the veracity of measures of heterogeneity is arguable; therefore, their interpretation should be made with caution^97^. Statistical heterogeneity was calculated with the *I*^2^ test for each outcome domain. Heterogeneity *I*^2^ ≥ 60% was regarded as substantial heterogeneity, in which case no pooled effect sizes are reported. Subgroup analyses were planned to explore possible sources of heterogeneity on population, duration of the intervention, delivery of the intervention, and presence of guidance.

### Reporting summary

Further information on research design is available in the Nature Research Reporting Summary linked to this article.

## Supplementary information

Supplementary material

Reporting Summary

## Data Availability

Data supporting the findings of this study are available within the respective articles cited in this review and from the corresponding author on reasonable request.

## Code availability

Meta-analyses were conducted using the R Studio packages Meta^100^ and Metafor^103^, and Review Manager^101,102^. The code is publicly available in the following github repository: https://github.com/MathiasHarrer/Doing-Meta-Analysis-in-R.
