# Supplementary material for: Culturally adapting internet- and mobile-based health promotion interventions might not be worth the effort: a systematic review and meta-analysis
Source: NPJ Digit Med. 2022 Mar 23;5:34. doi: 10.1038/s41746-022-00569-x (PMC8943001; doi:10.1038/s41746-022-00569-x)
Supplement: Supplementary file 1 — Supplementary material [file 41746_2022_569_MOESM1_ESM.pdf]

Supplementary Material

1 Changes to study protocol

1.1 Pre-defined sensitivity and subgroup analyses were not performed due to low number of included studies, n=9.

1.2 A search update included the additional database of Web of Science.

2 Supplementary Tables

2.1 Supplementary Table 1

Supplementary Table 1. Individual data points of the studies reporting continues outcomes

First author (Year)	IG (M)	IG (SD)	WL (M)	WL (SD)	IG (N)	WL (N)
Studies reporting continues outcomes of long-term effectiveness comparing to active control conditions						
Bender 2017	9524	773.06	7208	566.95	22	23
Kurth 2016	0.32	0.84	0.31	0.83	205	200
Larsen 2020	60.93	44.64	60.56	92.26	22	24
Marcus 2016	112.8	97.1	63.5	88.7	104	101
Fortmann 2017	31.9	5.4	32.1	6.6	50	58
Montag 2015	1.64	5.76	1.99	5.26	110	131
Studies reporting continues outcomes of short-term effectiveness comparing to active control conditions						
Bender 2017	7483	2415	6735	2363	22	23
Kurth 2016	0.42	0.71	0.37	0.73	220	209
Fortmann 2017	31.7	5.2	32	6.1	49	57
Montag 2015	0.98	2.48	1.94	3.74	91	97
Studies reporting continues outcomes of short-term effectiveness comparing to passive control conditions						
Brito Beck da Silva 2019	20.95	4.17	20.61	3.79	285	317
Duan 2017	557.51	417.51	499.19	470.3	88	54
Lau 2019	18.75	10.57	23.81	13.34	13	16

IG: intervention group, *WL*: waitlist, *M*: mean, *SD*: standard deviation
