# Supplementary material for: Culturally adapting internet- and mobile-based health promotion interventions might not be worth the effort: a systematic review and meta-analysis
Source: NPJ Digit Med. 2022 Mar 23;5:34. doi: 10.1038/s41746-022-00569-x (PMC8943001; doi:10.1038/s41746-022-00569-x)
Supplement: Supplementary file 2 — Reporting Summary [file 41746_2022_569_MOESM2_ESM.pdf]

nature portfolio

Corresponding author(s): Sumeyye BALCI
Last updated by author(s): Nov 3, 2021

Reporting Summary

Nature Portfolio wishes to improve the reproducibility of the work that we publish. This form provides structure for consistency and transparency in reporting. For further information on Nature Portfolio policies, see our Editorial Policies and the Editorial Policy Checklist.

Statistics

For all statistical analyses, confirm that the following items are present in the figure legend, table legend, main text, or Methods section.

n/a	Confirmed	
☐	☒	The exact sample size (n) for each experimental group/condition, given as a discrete number and unit of measurement
☒	☐	A statement on whether measurements were taken from distinct samples or whether the same sample was measured repeatedly
☐	☒	The statistical test(s) used AND whether they are one- or two-sided<br>*Only common tests should be described solely by name; describe more complex techniques in the Methods section.*
☒	☐	A description of all covariates tested
☒	☐	A description of any assumptions or corrections, such as tests of normality and adjustment for multiple comparisons
☐	☒	A full description of the statistical parameters including central tendency (e.g. means) or other basic estimates (e.g. regression coefficient) AND variation (e.g. standard deviation) or associated estimates of uncertainty (e.g. confidence intervals)
☐	☒	For null hypothesis testing, the test statistic (e.g. F, t, r) with confidence intervals, effect sizes, degrees of freedom and P value noted<br>*Give P values as exact values whenever suitable.*
☒	☐	For Bayesian analysis, information on the choice of priors and Markov chain Monte Carlo settings
☒	☐	For hierarchical and complex designs, identification of the appropriate level for tests and full reporting of outcomes
☐	☒	Estimates of effect sizes (e.g. Cohen's d, Pearson's r), indicating how they were calculated

Our web collection on statistics for biologists contains articles on many of the points above.

Software and code

Policy information about availability of computer code

Data collection	R Studio 4.0.1 with packages meta (v. 4.16-2) and metafor(2.4-0), SPSS v. 26, RevMan v. 5.3
Data analysis	R Studio 4.0.1 with packages meta (v. 4.16-2) and metafor(2.4-0), SPSS v. 26, RevMan v. 5.3

For manuscripts utilizing custom algorithms or software that are central to the research but not yet described in published literature, software must be made available to editors and reviewers. We strongly encourage code deposition in a community repository (e.g. GitHub). See the Nature Portfolio guidelines for submitting code & software for further information.

Data

Policy information about availability of data

All manuscripts must include a data availability statement. This statement should provide the following information, where applicable:

- Accession codes, unique identifiers, or web links for publicly available datasets
- A description of any restrictions on data availability
- For clinical datasets or third party data, please ensure that the statement adheres to our policy

Data supporting the findings of this study are available within the articles cited in this review and respective authors.

Field-specific reporting

Please select the one below that is the best fit for your research. If you are not sure, read the appropriate sections before making your selection.

☐ Life sciences ☒ Behavioural & social sciences ☐ Ecological, evolutionary & environmental sciences

For a reference copy of the document with all sections, see nature.com/documents/nr-reporting-summary-flat.pdf

Behavioural & social sciences study design

All studies must disclose on these points even when the disclosure is negative.

Study description	A meta-analysis and a systematic review on quantitative data from culturally adapted internet- and mobile-based health promotion interventions.
Research sample	Studies were included if they (1) were RCTs, (2) had no treatment, treatment as usual (TAU), placebo, waitlist, or active control conditions, (3) were delivered via the internet, (4) were culturally adapted for a population that differed from the original intervention's target group, (5) examined a health promotion intervention on healthy eating, physical activity, alcohol consumption, sexual health behavior and/or smoking cessation (6) reported one of the respective health promotion-specific outcomes: body mass index (BMI), time spent exercising, change in condom use, level of smoking, level of alcohol consumption, or one of the following generic outcomes: health-related quality of life and self-efficacy.
Sampling strategy	Adapted intervention studies were included if they had a sample, which differed in terms of cultural background from the original intervention's target group
Data collection	Data collection was done via sytematic searches in the following databases: Cochrane Central Register of Controlled Trials (CENTRAL), EbscoHost/MEDLINE, Ovid/Embase, EbscoHost/PsychINFO, and Web of Science. Title, Abstract, and Full-text screening are done via two independent researchers on Covidence website.
Timing	Initial search was conducted on 26. August 2019. A search update was conducted on 15. October 2020.
Data exclusions	Studies were excluded if they did not met the inclusion criteria.
Non-participation	No individual participant data were used.
Randomization	Only randomized controlled trials were included.

Reporting for specific materials, systems and methods

We require information from authors about some types of materials, experimental systems and methods used in many studies. Here, indicate whether each material, system or method listed is relevant to your study. If you are not sure if a list item applies to your research, read the appropriate section before selecting a response.

Materials & experimental systems

n/a	Involved in the study	
☒	☐	Antibodies
☒	☐	Eukaryotic cell lines
☒	☐	Palaeontology and archaeology
☒	☐	Animals and other organisms
☐	☒	Human research participants
☒	☐	Clinical data
☒	☐	Dual use research of concern

Methods

n/a	Involved in the study	
☒	☐	ChIP-seq
☒	☐	Flow cytometry
☒	☐	MRI-based neuroimaging

Human research participants

Policy information about studies involving human research participants

Population characteristics	See above.
Recruitment	Data collection was done via sytematic searches in the following databases: Cochrane Central Register of Controlled Trials (CENTRAL), EbscoHost/MEDLINE, Ovid/Embase, EbscoHost/PsychINFO, and Web of Science. Title, Abstract, and Full-text screening are done via two independent researchers on Covidence website.
Ethics oversight	No individual participant data was used in this review.

Note that full information on the approval of the study protocol must also be provided in the manuscript.
